# Executive Functions and Body Weight at Different Ages: A Preliminary Study

**DOI:** 10.3390/nu13041174

**Published:** 2021-04-02

**Authors:** Francesca Favieri, Eunice Chen, Maria Casagrande

**Affiliations:** 1 Department of Psychology, “Sapienza” University of Rome, 00185 Rome, Italy; 2 Department of Psychology, Temple University, Filadelfia, PA 19122, USA; Eunice.Chen@temple.edu; 3 Department of Dynamic and Clinical Psychology and Health Studies, “Sapienza” University of Rome, 00185 Rome, Italy

**Keywords:** overweight, executive functions, development, body weight

## Abstract

Recently, researchers have focused their attention on the role of cognitive processes on eating habits and body weight changes. Few studies have examined the relationship between the first stages of overweight and executive functions (EFs), excluding obesity conditions. This study is aimed to detect the involvement of the EFs and their predictive role on body mass index (BMI) in a sample of healthy individuals from childhood to young adulthood with a cross-sectional design. One-hundred and sixty-six healthy students were recruited from different Italian public schools: 46 children (age range: 7–11), 50 adolescents (age range: 15–18), 70 young adults (age range: 19–30). BMI was used to evaluate body weight and different EFs tasks were used to assess the EFs domains of inhibition, updating and shifting. After adjusting BMI for age, a hierarchical multiple linear regression was carried out for each EFs task. Pearson’s r correlations were reported for each of the age subgroups. Motor disinhibition was associated with greater BMI in the overall sample. Higher BMI was related to poorer set-shifting in adolescence and poorer motor inhibition in young adulthood, but higher BMI was not associated with EFs in childhood. Differences in the development of EFs over time may influence weight changes over time through different responses to food and eating behavior.

## 1. Introduction

Obesity is an important public health problem that increases the risk of chronic diseases (e.g., cardiovascular pathologies, type-2 diabetes, hypertension, fibromyalgia) and psychological problems (i.e., depression, anxiety, psychopathological symptomatology) [1]. Moreover, much data suggest that obesity is a risk factor for the development of neurocognitive decline, independently of age, education, general cognitive condition and other lifestyles or health variables [2,3,4,5,6].

The most recent European prevalence data reported that about 58 percent of the population over 18 years is affected by overweight, with a range between 45 (Tajikistan) and 67 percent (Turkey) [7]. This percentage is confirmed in Italy, where 58 percent of the population over 18 years report high body fat levels. The prevalence of overweight and obesity has also increased in younger people (i.e., children and adolescents) in Europe [8].

Usually, excessive body weight is strictly linked to a sedentary lifestyle and unhealthy eating habits [9]. However, obesity is multifactorial in terms of etiology. Many biological (e.g., genetics, prenatal and perinatal aspects, neuroendocrine and physical conditions, autonomic and neurological characteristics), environmental (e.g., socioeconomic status, cultural and social biases, food availability) and behavioral (eating pattern, sedentary lifestyles, caloric intake, daily activities) factors concur to determine obesity [10], from childhood [11] to the whole life span [12]. Recently researchers have focused their attention on the role of cognitive processes in eating habits and related body weight changes [6,13,14,15]. Some findings suggest that EFs (including inhibitory control, set-shifting and working memory; [16,17] may moderate the relationship between the intention and the act of eating, influencing the intake of food [2,18]. Many studies report an association between cognitive deficits (involving memory, learning, attention and executive functions) and excessive body weight, considering both overweight and obesity [13,14,19]. Different authors have developed preliminary conceptual models [2,20] to define the relationship between EFs and obesity. Some researchers argue that this association supports the idea that there is a genetic vulnerability to overweight expressed in cognitive functioning and alteration in brain morphology [21]. Other authors underlined a strong bidirectional relationship between cognitive impairment and increased body weight with an important role played by the executive functions (EFs; [2,22,23]). This last association is confirmed in people of different ages [22]. However, as highlighted by a recent systematic review and meta-analysis [24], this relationship is still unclear due to the limited number of studies that considered the different overweight stages separately.

Early association between obesity and EFs impairment was supported [6,25], specifically with regards to cognitive disinhibition [24], reported in children and adolescents with obesity. Deterioration in executive functioning from young adulthood and late adulthood has been reported [6]. However, there is a lack of consensus about the nature of the relationship between EFs and obesity and the evolution over the life span, especially in children and adolescents [22,26,27]. This limit would be ascribed to the well-known difficulty in tracing a clear trajectory in the development of EFs, regardless of their relationship to behavioral variables [28]. Generally, the studies on EFs reported a not uniform developmental process, due to the in-progress connections of the prefrontal brain areas during childhood, with full development in later childhood (e.g., inhibitory control; working memory) or early adulthood (e.g., shifting) of different EFs (for a review see [28].

To our knowledge, no study has been conducted in a sample that included an age range from childhood to young adulthood to assess the association between EFs and weight, considering the early stages of overweight. Most studies have focused on obesity and a few on the less severe but more frequently occurring problem of overweight in the absence of medical conditions [29,30]. This limit could be due to the indices adopted to assess weight status in young and adults. In both clinical and research fields, one of the most frequently adopted measures is the body mass index [31], which presents some limits in identifying the risk associated with excessive body weight, specifically in younger samples [32]. Some authors have attempted to be overcome this limit by adopting reference charts referring to the general population [33]. However, these limitations have prevented studies from including populations of children and adolescents.

This study examines the association between weight status and EFs in a sample of children, adolescents and young adults recruited from the general population, who had a healthy weight and overweight but did not meet the criteria for obesity or eating disorders. In line with previous studies, we hypothesized a general association between increased body weight and worse performance in executive tasks assessing different executive functions (i.e., inhibition, working memory and shifting), independently of age [22]. This hypothesis is in line with Miyake’s results on EFs that identify a correlation between different EFs although they are clearly separable [17].

Moreover, according to developmental models of EFs across the lifespan [34], which reported a stronger development of EFs in the transition from childhood to adolescence, with stabilization from middle adolescence to adulthood and a subsequent deterioration in the elderly [35], we expected different components of EFs could be related to BMI status when considering children, adolescents and young adults.

## 2. Materials and Methods

### 2.1. Participants

One-hundred and sixty-six students (65 males and 101 females; mean age: 17.06 years SD = 6.55) were recruited from different Italian public schools. Specifically, 46 children were from first grade (age range: 7–11; 22 males, 24 females; mean age: 7.98 years SD = 0.88), 50 adolescents from the high school (age range: 15–18; 15 males, 35 females; mean age: 16.64 years SD = 0.75) and 70 young adults from college (age range: 19–30; 31 males, 42 females; mean age: 23.32 years SD = 2.57).

The participants were included in the study if they did not present an eating disorders diagnosis, severe obesity, developmental disorders, chronic medical diseases, or any psychopathological condition (e.g., anxiety, depression). Because of the tasks administered, normal or corrected-to-normal vision and the absence of color blindness were required. All participants were selected on a voluntary basis. In the case of children and adolescents, a preliminary presentation of the research design was made in schools to both children and parents and consents were collected for participation in the study. In addition, evaluations were also carried out to participants who had any of the exclusion criteria included in the study (respectively, 5 children; 3 adolescents and 1 young adult), but the data were not included in the data analysis set. This recruitment process was done in accordance with the guidance of the local ethics committee.

Fifty-nine participants (20 males, 39 females) were included who were in the overweight category and 107 participants (49 males, 62 females) who were in the normal weight category according to WHO weight classification both for children and adolescents and adults [31].

Table 1 shows the main characteristics of the participants considering age and weight classification.

### 2.2. Apparatus

A digital balance was used to assess each participant’s weight (Kg) and a wall-mounted anthropometer was used to measure the height (m). These two measures allowed the body mass index (BMI) to be computed by dividing weight by height (meters squared). The WHO criteria were adopted, using the growth charts for children and adolescents [31] and the BMI classification for young adults [31].

A tape measure was adopted to measure waist and hip circumferences, to calculate the waist-to-height ratio (W/Hr; [36]) and body adiposity index (BAI = ((hip circumference)/((height)1.5) − 18)); [37]). A digital sphygmomanometer was used to measure systolic and diastolic blood pressure.

### 2.3. Apparatus

Demographic information

A semi-structured interview was adopted to collect each participant’s main demographic information (gender, age, level of education) and medical and clinical history. For children and adolescents under 18 years of age, the interview was conducted by one of the parents.

Executive functions

According to Diamond’s model [16], executive functions were assessed in all age groups; standardized tasks previously adopted in childhood, adolescence and adulthood were used.

Apparatus: computerized versions of the Stroop task, Go/No-go task and n back task were presented throughout the E-Prime 2.0 software on an Intel Core i5 PC and displayed on a 17-inch color screen. A standard computer keyboard collected the responses.

Cognitive Inhibition:

- The Stroop task [38] is one of the most adopted instruments for assessing executive functions, specifically cognitive inhibition and interference control.

**Figure 1:**
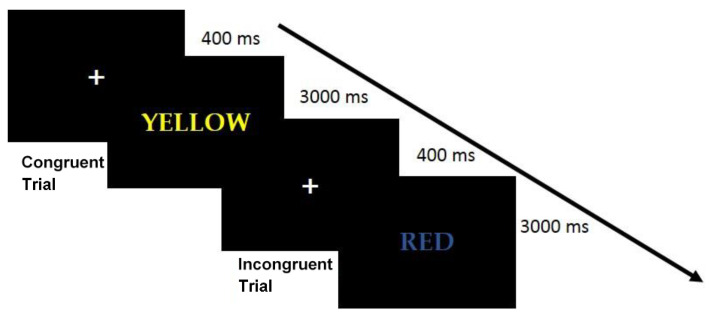
Example of stimuli and procedure of the Stroop Task.

Visual Stimuli and procedure: the task provided the administration of target stimuli consisted of colored words (Font: Courier New; Font size: 60; colors: yellow, red, blue, green) referred semantically to the colors YELLOW, RED, BLUE, GREEN. Each word could be presented with the ink color related to its semantic meaning (Congruent Condition, e.g., RED wrote in red ink) or with another color (Incongruent Condition, e.g., RED wrote in blue ink). As quickly and accurately as possible, the task required to press the key corresponding to the ink color (key “A” = red; Key “S” = green; Key “K” = blue; Key “L” = yellow). The experiment was introduced by a practice block of 15 trials with feedback about their correct execution. Afterward, a block of 120 randomly presented trials (60 Congruent and 60 Incongruent) was presented. An initial fixation cross (duration: 400 ms) was presented before each trial. The target stimulus remained on the screen for 3000 ms or until the participant’s response. To ensure good validity of the measures, in line with the literature on the task, all participants who achieved an accuracy greater than 65 percent were included in the study. Moreover, trials with reaction times over 200 ms and correct trials were considered to calculate each participant’s mean Reaction Time (RT) for congruent and incongruent conditions and the Stroop Effect (mean RTs Incongruent Trials—mean RTs Congruent Trials) was computed. Procedure and stimuli are shown in Figure 1.

- The Go/No-Go Task [39] allows assessing motor inhibition, i.e., the ability to control an inadequate motor response.

**Figure 2:**
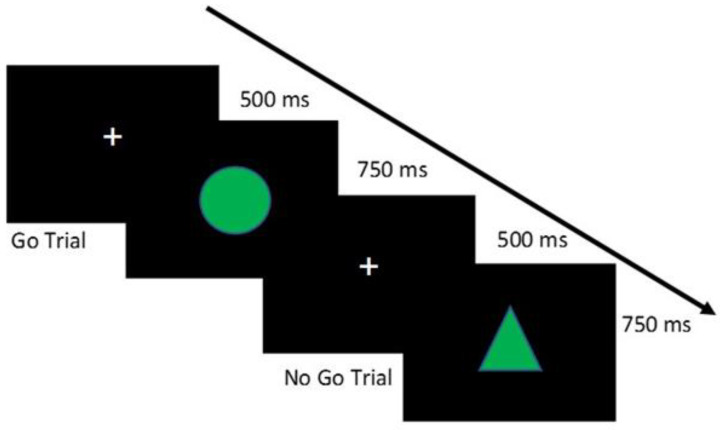
Example of stimuli and procedure of the Go/No-Go Task.

Visual Stimuli and procedure: the stimuli included two geometric shapes of 960 × 720 pixels each, placed in the center of the screen with a black background. The Go stimulus was a green circle and the No-Go stimulus was a green triangle. The participant was required to keep his gaze fixed in the center of the screen for the duration of the experiment. The initial screen with a fixation cross (duration: 500 ms) was followed by the presentation of target stimuli (Go) and non-target stimuli (No-Go), in a randomized way considering three, four, or five Go trials for each No-Go trial. Each stimulus remained in the center of the screen for 750 ms or until the participant’s response. The task required to press the left mouse key as quickly as possible when the green circle appeared in the center of the screen. When the green triangle appeared, the participant had to wait for the disappearance of the stimulus. The task involved a total of 100 trials divided into two blocks of 50 trials each. A practice block of 12 trials, with feedback on correctness, was presented at the beginning of the experiment. The inappropriate responses to “no-go” stimuli were summed to define the numbers of False Alarms, considered the inhibition motor component. An accuracy greater than 50% was accepted for including the participant in the analysis. Procedure and stimuli are shown in Figure 2.

Working Memory:

- The n-back task [40] is largely adopted to assess working memory.

**Figure 3:**
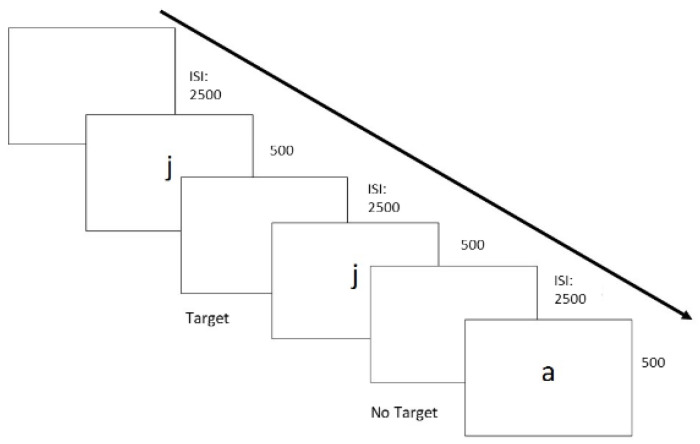
Example of stimuli and procedure of the N-Back Task (1-Back Version).

Visual Stimuli and procedure: the stimuli included some alphabetical letters presented in the middle of the screen (Font: Palatino Linotype; Font size: 30) with a white background. Two sessions of the task were presented in sequence: the one- and the two-back. A sequence of stimuli one-by-one (duration: 500 ms), followed by a blank screen (ISI: 2500 ms), was presented. In the one-back session, the participant evaluated whether each stimulus was the same as the previous stimulus (Target) or different from the previous stimulus (Non-Target). In the two-back session, the participant had to indicate whether the stimulus was the same or different from the stimulus presented in the two previous trials. The responses were collected by pressing the key “X” for the target stimuli and the key “M” for the non-target stimuli. The overall task includes 40 trials for the one-back block and 40 trials for the two-back blocks. In each block the 30% of the trials were Target. The percentage of accuracy for both one-back and two-back procedures was considered a measure of working memory. According to Pelegrina et al. [41] who reported heterogeneous results in children at the n-back tasks but confirmed the validity of using the task from school-age children, over the 20 percent was adopted as the lowest accuracy. Procedure and stimuli are shown in Figure 3.

Set-shifting:

- The Wisconsin Card Sorting Test (WCST, [42]), a computerized version of the WCST was adopted to assess cognitive flexibility. The test requires the participant to match some cards according to specific characteristics of four Stimulus Cards.

Visual Stimuli and procedure: two sets of 64 cards were presented. There were four possible suits in the cards (i.e., stars, crosses, circles, triangles), they can be of four possible colors (i.e., red, yellow, green, blue) and each card can have a different number of suits, from 1 to 4. The cards are sorted by reference to four stimulus cards with one red triangle, two green stars, three yellow crosses and four blue circles. The participant has to place a single card of the deck in correspondence with one of the four initial stimulus cards. At each participant’s choice, the experimenter provides feedback on the correctness of the answer to allow the participant to infer the adopted criterion (i.e., color, shape, number, respectively) to sort the cards through this feedback ignoring the other criteria. This procedure continues until the participant makes ten correct choices. Then, the correct answer criterion changes. The procedure continues until the two decks of cards are completed. Different indices can be extrapolated from the test. In this study, the global score ([card administered—(completed categories*10)] with lower scores as indices of better performance) and perseveration errors (as an index of difficulty in set-shifting the procedure to complete the task).

### 2.4. General Procedure

The research was conducted according to the Helsinki Declaration and the Local Ethics Committee (Department of Dynamic and Clinical Psychology and Health Studies —“Sapienza” the University of Rome; protocol n. 0000450-15/04/2019) approved it. The written informed consent was explained and signature was sought before the evaluation, with parents consenting on behalf of their child and both participants and parents consenting on behalf of adolescents.

Each participant was individually tested in a silent, dimly illuminated room with a comfortable temperature and the procedure was explained prior to the in-person interview. Researchers trained in the use of the e-prime software administered the tasks and were present throughout the experimental procedure. All the evaluators were attending a doctoral program or had a Ph.D. degree in psychology and cognitive science, which made them qualified to implement experiments and the administration of cognitive tasks. Participants indicated their current hunger levels on a visual-analogue scale (0–100 mm) and then completed the experimental tasks. For each computerized version of the cognitive tasks, a practice block was included to allow the participant to become familiar with the task. Finally, weight, height and blood pressure were measured.

### 2.5. Data Analysis

Data are reported as means and standard deviations for each age group (children, adolescents and young adults) and the weight condition (normal weight, overweight). Two-way ANOVAs were carried out to control the differences between groups considering each variable. Furthermore, a hierarchical multiple linear regression was carried out on the whole sample to evaluate the role of executive functions on BMI variation, considering the task assessing the three EFs of Diamond’s model (inhibition, working memory and shifting) in line with the aim of the study. To control for the greater impact of high BMI in younger ages, BMI to age ratio was calculated. To control the role of age in the relationship among variables, age was added as a covariate in the regression model. The 3 steps assessed in the hierarchical multiple linear regression model were: (1) cognitive inhibition: including false alarms on the Go/No-Go task and Stroop effect (RT) indices; (2) working memory: including accuracy at the one and two-back; (3) shifting: including global score and perseverations on the WCST. To assess the association between weight condition (BMI, waist-to-height ratio, Body adiposity index) and executive functioning in the different age groups, sub-groups linear Pearson’s r correlation was calculated and Benjamini–Hochberg correction for the *p*-value was included.

## 3. Results

### 3.1. General Data of the Sample

Table 1 shows the sample’s main characteristics (means and std.dev.) classified according to age and weight condition. In addition, the performance of the groups in executive tasks is shown.

### 3.2. Regression Analysis

The hierarchical regression model adjusted for age was significant (see Table 2). The best model considered the inhibition indices (Stroop Effect and False Alarms) (F_2,160_ = 5.49; *p* = 0.005; Adjusted R2 = 0.73). Specifically, considering the predictors of the models, only False Alarms on the Go/No-Go task were related to increased age-adjusted BMI. 

Table 3 shows intercorrelations of cognitive measures.

### 3.3. Correlation Analysis

Table 4, Table 5 and Table 6 reported linear Pearson’s r correlation in the three different age groups considering the weight indices of BMI, waist-to-height ratio, Body adiposity index and self-reported hunger before the EF task administration.

Specifically, in children, no significant correlations were found (Table 4).

In adolescents, BMI was correlated with worse performance in WCST considering both global score (r = 0.43; *p* = 0.02) and perseveration (r = 0.41; *p* = 0.03). Moreover, positive linear correlations were reported between self-reported hunger and the false alarms index (r= 0.55; *p* = 0.003) and lower accuracy on the 1-back task (r = −0.39; *p* = 0.05) (Table 5).

In the young adults’ group, the only significant correlation was between BMI and false alarms in the Go/No-Go task (r = 0.28; *p* = 0.05) (Table 6).

## 4. Discussion

Motor disinhibition was associated with greater BMI in the overall sample. In adolescence, higher BMI was associated with poorer set-shifting, while in young adulthood, higher BMI was associated with poorer motor inhibition; however, no associations with higher BMI and EFs were found in childhood.

This study examined associations between weight and executive functioning, according to Diamond’s tripartite model [16], which organized the EFs in three main domains: working memory updating, set-shifting and cognitive inhibition. Moreover, according to Miyake’s study [17], a correlation between the domains and a possibility for them to be evaluated independently, the role of the EFs was interpreted in their association with body weight change. In the whole sample, more False Alarms on the Go/No-Go task were related to increased age-adjusted BMI, suggesting greater motor disinhibition is associated with greater BMI, independently from age. Other studies have shown that higher age-adjusted BMI is related to poorer performance on a task aimed to assess motor inhibitory control [43], with similar results seen in a child sample in the overweight range [44]. Other studies have shown a relationship between obesity and motor disinhibition in adolescents [45,46]. We did not find evidence of an association between age-adjusted BMI and performance on the Stroop task that assesses cognitive inhibition. The discrepancy in findings between the two inhibitory control measures may be explained by different neural networks being implicated in motor and cognitive inhibition [47]. Furthermore, it fits with a meta-analysis showing that greater BMI is associated with supplementary motor area differences in response to palatable food consumption [48]. Dissociation of whether motor inhibitory control is due to overweight status or difficulties inhibiting behavior that predates weight gain is needed.

Different patterns between weight status and EFs emerged across the three different age groups. In the current study, there were no associations between EFs and higher BMI in children, which may reflect the fact that the neural structures associated with EF develop later [28,49]. This assumption could be supported by the general lower performance reported in the children’s group in the cognitive tasks. In adolescents in the current study, poorer set-shifting, as assessed by WCST, was associated with higher BMI. While these findings fit with previous studies of adolescents with obesity [50] and eating disorders [51,52] who showed worse cognitive flexibility and set-shifting compared to adolescents with a healthy weight, it did not agree with a previous study with overweight adolescents [53]. In the current study, in young adults, higher BMI was associated with poorer motor inhibition, as in the broader findings that considered obesity conditions [54,55], confirming a similar pattern also in less severe overweight.

These results should be considered preliminary. The current study was cross-sectional and not longitudinal and future studies are needed to examine the relationship between EFs and BMI across the lifespan in the same individuals. This would control the limitations of an independent-group comparison to test the hypotheses of interest in the study. In addition, a longitudinal design could allow, despite the difficulties in its application, us to understand the interaction of variables during the development. Moreover, this appears relevant considering the evolution of the relationship of BMI–age, which can be characterized by a more or less significant increase or decrease, especially in mild overweight. Therefore, from this perspective, the study is a starting point to methodologically set up future studies aimed at assessing the association between executive functions and body weight at different stages of life. As previously reported, this study, like all others on the topic, is characterized by the limit of the BMI adoption, because it is an indirect measure of body fat and may not reflect and differentiate changes in body fat and muscle mass [56]. This may not have shown a strong association between executive functions and body condition, as the same index could be reported for individuals with different body shapes associated with healthy or maladaptive lifestyle habits. The sample size was small and future studies are needed that include a group of middle-aged adults. Moreover, these preliminary results should be deepened through other approaches, including statistical approaches, to control how age acts as a moderator of the relationship, at different developmental stages. The replicability of this study is also important to show that these results were not due to random chance or the characteristics of the tasks generally easier for young adults than others group and characterized in some cases by low accuracy in younger. Moreover, the absence of cut-off did not allow to verify a claimed executive impairment. Even a possible selection bias, which depended on voluntary participation in the study and the recruitment only in some schools located in the Roman territory and the limited sample size represent a limitation for the generalizability of the results.

Nonetheless, the current study extends existent findings by examining the effect of overweight, rather than obesity, in different age groups including children in a physically healthy sample. The current study also examined a range of EFs typically not examined in studies of children.

## 5. Conclusions

This study supports the association between increased BMI and EFs in adolescence and young adulthood in a sample ranging from a healthy weight to overweight, who were physically healthy. Higher BMI was associated with poorer set-shifting in adolescence and poorer motor inhibition in young adulthood. Motor disinhibition was associated with greater BMI in the overall sample. However, we cannot speak of executive impairment, but rather of an association between better (or higher) and worse (or lower) functioning and body weight changes. While this is a preliminary cross-sectional study, it suggests that executive functioning problems are associated with greater BMI in adolescence and young adulthood even in a healthy sample in the healthy weight to the overweight range, representing the majority of the population in this age-group. Longitudinal investigations of executive functioning and their relationship with weight and related indices in the same individuals are needed from childhood through adulthood.

## Figures and Tables

**Table 1:** Characteristics of participants and performances in the tasks for the assessment of the executive functions.

	Children		Adolescents		Young Adults				
	Normal Weight	Overweight	Normal Weight	Overweight	Normal Weight	Overweight	F_2,160_	*p*	η_p_^2^ *
N (m/f)	31 (17/14)	15 (5/10)	38 (13/25)	12 (2/10)	38 (15/23)	32 (13/19)			
Age	8.00 (0.93)	7.93 (0.80)	16.71 (0.77)	16.42 (0.67)	22.39 (2.28)	24.44 (2.47)	8.12	0.001	0.10
Years of Education	2.9 (0.75)	2.93 (0.80)	11.71 (0.77)	11.42 (0.67)	15.89 (1.33)	16.84 (1.22)	5.36	0.01	0.06
Weight (kg)	27.79 (5.35)	38.24 (7.96)	58.10 (7.38)	75.33 (11.36)	61.45 (8.78)	80.03 (15.18)	2.39	0.10	0.03
Height (m)	1.32 (0.08)	1.35 (0.07)	1.68 (0.07)	1.68 (0.11)	1.69 (0.09)	1.70 (0.08)	<1	0.59	0.01
BMI	15.83 (1.70)	20.74 (3.00)	20.46 (1.97)	26.74 (3.30)	21.34 (1.74)	27.35 (3.05)	<1	0.37	0.01
BMI-to-age ratio	1.99 (0.23)	2.64 (0.43)	1.23 (0.13)	1.63 (0.22)	0.97 (0.13)	1.11 (0.13)	20.78	0.001	0.21
Waist-to-height ratio	0.45 (0.04)	0.50 (0.06)	0.40 (0.08)	0.47 (0.04)	0.43 (0.04)	0.50 (0.10)	<1	0.72	0.004
Body Adiposity Index	24.56 (4.52)	29.61 (6.43)	20.48 (8.59)	26.18 (5.67)	21.23 (3.13)	26.82 (9.79)	<1	0.97	0.001
Systolic Blood Pressure	108.52 (17.71)	113.65 (7.55)	104.75 (12.36)	111.41 (12.48)	114.42 (10.68)	119.39 (11.46)	<1	0.95	0.001
Diastolic Blood Pressure	67.74 (11.14)	76.25 (10.73)	68.16 (7.13)	72.54 (7.74)	69.30 (8.30)	74.70 (6.84)	<1	0.61	0.01
Hunger (0–100)	50.80 (33.83)	37.17 (27.05)	40.28 (34.63)	45.83 (31.48)	61.06 (26.65)	45.13 (33.63)	1.40	0.25	0.01
Cognitive Inhibition									
Stroop Effect (Stroop Task: reaction times)	91.96 (81.22)	108.23 (82.46)	66.59 (44.94)	103.61 (63.78)	91.91 (60.18)	98.24 (69.83)	<1	0.53	0.01
False Alarms (Go-No Task)	7.74 (4.15)	8.83 (5.20)	4.66 (3.53)	5.67 (4.08)	3.97 (3.14)	4.55 (2.79)	<1	0.92	0.001
Working memory									
1-back task accuracy (%)	0.70 (0.28)	0.72 (0.25)	0.93 (0.10)	0.88 (0.13)	0.94 (0.10)	0.93 (0.11)	<1	0.60	0.01
2-back task accuracy (%)	0.50 (0.27)	0.51 (0.24)	0.67 (0.22)	0.71 (0.21)	0.79 (0.17)	0.82 (0.21)	<1	0.98	0.002
Set-shifting									
Global Score (WCST)	33.63 (20.98)	37.47 (16.90)	21.13 (16.62)	25.17 (19.09)	16.63 (16.01)	13.16 (10.85)	<1	0.70	0.004
Perseveration (WCST)	10.50 (9.05)	11.93 (10.44)	6.03 (3.74)	7.92 (4.91)	4.87 (4.21)	4.38 (3.59)	<1	0.61	0.01

* partial eta square.

**Table 2:** Hierarchical regression model considering the BMI-to-age ratio as dependent variable (adjusted for age).

Models		B	Standard Error	Beta	t	Sign. (*p*=)	95% CI Lower	95% CI Upper	Zero-Order Correlation	% of Explenation
1	Stroop Effect	0.00	0.000	0.04	0.99	0.33	0.0	0.001	0.10	72
	False Alarms	**0.01**	**0.006**	**0.14**	**2.94**	**0.004**	**0.006**	**0.03**	**0.48**	
2	Stroop Effect	0.0	0.000	0.04	0.93	0.35	0.0	0.001	0.10	73
	False Alarms	**0.02**	**0.007**	**0.14**	**2.94**	**0.004**	**0.006**	**0.03**	**0.48**	
	1-back task accuracy	−0.17	0.145	−0.06	−1.14	0.25	−0.45	0.12	−0.42	
	2-back task accuracy	0.16	0.115	0.07	1.35	0.18	−0.07	0.38	−0.41	
3	Stroop Effect	0.0	0.000	0.04	0.88	0.38	0.0	0.001	0.10	73
	False Alarms	**0.02**	**0.007**	**0.14**	**2.92**	**0.004**	**0.006**	**0.03**	**0.48**	
	1-back task accuracy	−0.14	0.148	−0.05	−0.96	0.34	−0.43	0.15	−0.42	
	2-back task accuracy	0.15	0.116	0.07	1.33	0.19	−0.07	0.38	−0.41	
	Global Score (WCST)	0.002	0.002	0.06	0.91	0.36	−0.002	0.006	0.42	
	Perseveration (WCST)	−0.001	0.01	−0.01	−0.14	0.89	−0.012	0.01	0.36	

In bold: significant results.

**Table 3:** Intercorrelations of cognitive measures included in the regression model.

	Age-Adjusted BMI	Stroop Effect	False Alarms	1-Back Task	2-Back Task	Global Score WCST	Perseveration WCST
Stroop Effect	0.08 *p* = 0.20	1.00					
False Alarms	0.48 *p* = 0.0001	0.17 *p* = 0.01	1.00				
1-back task	−0.42 *p* = 0.0001	−0.07 *p* = 0.15	−0.32 *p* = 0.0001	1.00			
2-back task	−0.41 *p* = 0.0001	−0.03 *p* = 0.20	−0.32 *p* = 0.0001	0.54 *p* = 0.0001	1.00		
Global Score (WCST)	0.42 *p* = 0.0001	0.06 *p* = 0.20	0.19 *p* = 0.01	−0.32 *p* = 0.0001	−0.27 *p* = 0.0001	1.00	
Perseveration (WCST)	0.36 *p* = 0.0001	0.12 *p* = 0.10	0.13 *p* = 0.10	−0.25 *p* = 0.0001	−0.29 *p* = 0.0001	0.74 *p* = 0.0001	1.00

**Table 4:** Pearson’s r correlations in children.

		Stroop Effect (Stroop Task)	False Alarms (Go/No-Go Task)	1-Back Accuracy	2-Back Accuracy	Global Score (WCST)	Perseveration (WCST)
BMI	r	0.07	0.15	0.15	0.21	−0.01	0.02
	*p*	0.60	0.37	0.37	0.20	0.98	0.88
waist-to-height	r	−0.04	−0.05	0.28	0.24	0.04	−0.03
	*p*	0.82	0.75	0.08	0.14	0.83	0.84
BAI	r	0.16	0.18	0.24	0.21	−0.10	−0.19
	*p*	0.32	0.27	0.13	0.19	0.55	0.23
Self-reported hunger	r	−0.03	−0.13	−0.17	−0.20	0.13	−0.02
	*p*	0.86	0.42	0.30	0.24	0.41	0.91

Adjusted critical *p*-value (Benjamini–Hochberg correction) = 0.01.

**Table 5:** Pearson’s r correlations in adolescents.

		Stroop Effect (Stroop Task)	False Alarms (Go-Nogo Task)	1-Back Accuracy	2-Back Accuracy	Global Score (WCST)	Perseveration (WCST)
BMI	r	−0.05	−0.28	−0.001	0.11	**0.43**	**0.41**
	*p*	0.81	0.16	0.98	0.60	**0.02**	**0.03**
waist-to-height	r	0.06	0.12	−0.08	0.04	0.30	0.29
	*p*	0.75	0.55	0.69	0.86	0.13	0.14
BAI	r	0.02	0.12	−0.03	−0.01	0.24	0.22
	*p*	0.90	0.54	0.88	0.94	0.24	0.27
Self-reported hunger	r	0.18	**0.55**	**−0.39**	−0.06	−0.11	−0.13
	*p*	0.38	**0.003**	**0.05**	0.76	0.57	0.52

Adjusted critical *p*-value (Benjamini–Hochberg correction) = 0.03. In bold: significant results.

**Table 6:** Pearson’s r correlation in young adults.

		Stroop Effect (Stroop Task)	False Alarms (Go-Nogo Task)	1-Back Accuracy	2-Back Accuracy	Global Score (WCST)	Perseveration (WCST)
BMI	r	0.05	0.28	−0.23	0.14	−0.16	−0.18
	*p*	0.75	0.05	0.10	0.33	0.25	0.20
waist-to-height	r	−0.07	0.13	−0.10	0.05	−0.04	−0.05
	*p*	0.64	0.36	0.48	0.74	0.79	0.73
BAI	r	0.04	0.14	−0.15	0.08	0.07	0.03
	*p*	0.79	0.34	0.30	0.58	0.61	0.81
Self-reported hunger	r	0.01	0.06	−0.12	−0.10	0.13	0.14
	*p*	0.92	0.66	0.41	0.51	0.35	0.33

Adjusted critical *p*-value (Benjamini–Hochberg correction) = 0.01. In bold: significant results.

## Data Availability

The data presented in this study are available on request from the corresponding author.
